# *In vivo* measurement of mitochondrial ROS production in mouse models of photoreceptor degeneration

**DOI:** 10.1016/j.rbc.2023.100007

**Published:** 2023-12

**Authors:** Katja E. Menger, Angela Logan, Ulrich F.O. Luhmann, Alexander J. Smith, Alan F. Wright, Robin R. Ali, Michael P. Murphy

**Affiliations:** a UCL Institute of Ophthalmology, Bath St, London, EC1V 9EL, UK; b MRC-Mitochondrial Biology Unit, The Keith Peters Building, University of Cambridge, Cambridge, CB2 0XY, UK; c MRC Human Genetics Unit, Western General Hospital, University of Edinburgh, Edinburgh, EH4 2XU, UK

**Keywords:** Mitochondria: hydrogen peroxide, Retinitis pigmentosa: MitoB, Photoreceptor

## Abstract

Retinitis pigmentosa (RP) is a disease characterised by photoreceptor cell death. It can be initiated by mutations in a number of different genes, primarily affecting rods, which will die first, resulting in loss of night vision. The secondary death of cones then leads to loss of visual acuity and blindness. We set out to investigate whether increased mitochondrial reactive oxygen species (ROS) formation, plays a role in this sequential photoreceptor degeneration. To do this we measured mitochondrial H_2_O_2_ production within mouse eyes *in vivo* using the mass spectrometric probe MitoB. We found higher levels of mitochondrial ROS that preceded photoreceptor loss in four mouse models of RP: *Pde6b*^*rd1/rd1*^*; Prhp2*^*rds/rds*^*; RPGR*^*−/−*^*; Cln6*^*nclf*^. In contrast, there was no increase in mitochondrial ROS in loss of function models of vision loss (*GNAT*^*−/−*^*, OGC*), or where vision loss was not due to photoreceptor death (*Cln3*). Upregulation of *Nrf2* transcriptional activity with dimethylfumarate (DMF) lowered mitochondrial ROS in *RPGR*^*−/−*^ mice. These findings have important implications for the mechanism and treatment of RP.

## 1 Introduction

Retinitis pigmentosa (RP) is a heterogeneous inherited retinopathy disease caused by defects in at least 40 genes that can be autosomal or X-linked, and can present in recessive and dominant forms [1]. RP is characterised by the loss of photoreceptors from the retina, with rods typically affected first [2]. As rod photoreceptor cell death progresses, genetically unaffected cones begin to die off as well, leading to blindness. The reasons why cone cell death follows rod cell death are not well understood. Hypotheses include cessation of trophic factor release by rods, toxin release upon rod cell death, starvation of cones, the loss of structural support, or decreased rod oxygen consumption leading to hyperoxia and an increase in cone oxidative stress [[3], [4], [5], [6], [7], [8], [9]]. Here, we explore this last possibility. Photoreceptors are among the most metabolically active cells [[10], [11], [12], [13], [14]], consequently they contain a large number of mitochondria and have high oxygen consumption. However, to prevent blood cells disturbing the light path to the retina, oxygen is supplied by diffusion from the choroidal vasculature and thus cannot be regulated by demands of the outer retina. When photoreceptors die in large numbers, as in RP, the demand drops, and the oxygen tension and oxidative damage in the outer retinal layer increase [[15], [16], [17], [18], [19], [20], [21]]. As elevated oxygen tension increases mitochondrial H_2_O_2_ formation and oxidative damage [[22], [23], [24]], we hypothesised that the elevated local oxygen concentration after rod cell death may impact on cone cell viability by increasing mitochondrial H_2_O_2_ formation.

**Fig. 1:**
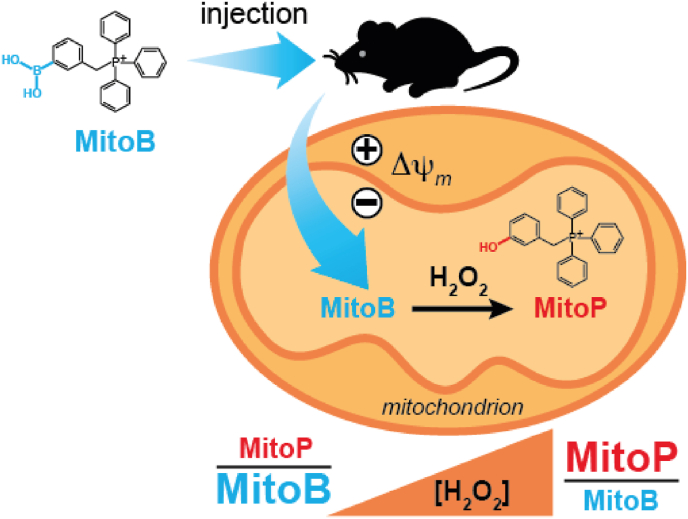
An illustration of the uptake of MitoB into the mitochondria of cells within tissues *in vivo* following injection, driven by the mitochondrial membrane potential. Within mitochondria the MitoB can be converted into MitoP by the action of H_2_O_2_ or ONOO^−^. The ratio of MitoP to MitoB can be readily determined by extraction of the tissue followed by analysis by LC-MS/MS. Thus, the MitoP/MitoP ratio provides a useful indication of relative levels of mitochondrial ROS *in vivo*.

To test whether elevated mitochondrial oxidative stress plays a role in photoreceptor degeneration it is vital to assess ROS production *in vivo*, as changes in oxygen levels during *ex vivo* measurements render these assessments uninformative. Therefore, we used the mitochondria-targeted mass spectrometric probe, MitoB, which can be injected intravitreally and then is slowly converted by mitochondrial H_2_O_2_ to MitoP over time [25] (Fig. 1). MitoB does not react directly with superoxide [26], but it does react rapidly with peroxynitrite to form MitoP [27], thus the conversion of MitoB to MitoP cannot be fully assigned to H_2_O_2_ unless a contribution from peroxynitrite production was eliminated. As this was not done here we cannot discount a contribution from peroxynitrite production to MitoB oxidation and thus here we use MitoB to assess changes in general mitochondrial ROS.

The extent of the conversion of MitoB to MitoP *in vivo* over time, measured ratiometrically as the MitoP/MitoB ratio, can then be determined by isolation of the tissue, followed by extraction and quantification of MitoP and MitoB by LC-MS/MS [25,26]. We used this approach to measure mitochondrial ROS levels *in vivo* within the eyes of both wild type mice of various ages as well as models of fast and slow photoreceptor degeneration and compared this with other models in which sight loss is caused by other factors. These models include: *Pde6b*^*rd1/rd1*^(rd1) which shows a loss of rod photoreceptors within the first 3 weeks of life, followed by subsequent cone loss, which is complete by 3 months; *Prph2*^*rds/rds*^ (rds) which has later onset loss of rod photoreceptors; *RPGR*^*−/−*^ which shows the slowest rate of degeneration [[28], [29], [30]]. We also assessed *Cln6*^*nclf*^ mice, a model of a lysosomal storage disorder that leads to neurodegeneration in both brain and retina and which shows photoreceptor degeneration, consistent with a rod-cone dystrophy [31]. We compared these with three other models which show negligible or very low levels of photoreceptor degeneration over the life-span of the mouse. *Gnat1*^*−/−*^, a model for stationary night blindness; *Opn4*^*−/−*^ *Gnat1*^*−/−*^ *Cnga3*^*−/−*^ (OGC) which lacks all light perception without substantive cell loss [28,29]; and Cln3^Δex7/8^, which predominantly shows loss of bipolar cells [32].

We found an elevation in mitochondrial H_2_O_2_ within the eyes of mouse models of RP, but not in the others. This is consistent with rod cell photoreceptor death subsequently affecting cone viability through local hyperoxia elevating mitochondrial ROS production and oxidative damage.

## 2 Materials and methods

### 2.1 Animals

Mice were used with institutional ethical approval and under a United Kingdom Home Office Project license and personal license. All procedures were performed in accordance with the Association for Research in Vision and Ophthalmology Statement for the Use of Animals in Ophthalmic and Vision Research. The following mice were used: C57Bl6/J, *Pde6B*^*rd1/rd1*^ (rd1), *Prph2*^*rds/rds*^ (rds), *Cln6*^*nclf*^, *Rpgr*^*−/−*^ [30]*, Cln3*^*Δex7/8*^ [32]*, Gnat1*^−/−^, *Opn4*^*−/−*^ *Gnat1*^*−/−*^ *Cnga3*^*−/−*^ (OGC on C57Bl6/129 mixed background) [28,29]. Mice were housed in the animal facility at University College London and bred for the relevant ages. They were housed at a 12 h/12 h light dark cycle with food and water ad lib.

### 2.2 Measurement of ROS formation

Mice were anaesthetised and MitoB (500 μM, 1 μl) was injected into the vitreous humour using a Hamilton syringe with a 34 ga needle. Anaesthesia was reversed and mice were sacrificed 9 h post MitoB injection. Eyes were removed, snap-frozen in liquid nitrogen and stored at −80 °C to prevent further reaction of MitoB to MitoP.

MitoB and MitoP were extracted from the tissue as previously described [32,33]. Briefly, the eye was homogenised at 4 °C in 100% acetonitrile (ACN) with 0.1% (v/v) formic acid (FA). After pelleting the supernatant was transferred to a fresh tube and the pellet was re-extracted with a second volume of 100% ACN and 0.1% (v/v) FA, and the new supernatant was combined with the first extraction. Internal standard of deuterated MitoB and MitoP was added to the supernatant and stored at 4 °C for 30 min. Protein precipitate was pelleted and the supernatant filtered and subsequently dried in a SpeedVac. Dried samples were resuspended in 20% (v/v) ACN, 0.1% (v/v) FA, any potential precipitate was pelleted and 200 μl of the supernatant were stored at 4 °C until LC-MS/MS analysis, which was done as previously described [25,26].

Samples were assessed by LC-MS/MS using an I-class Acquity LC attached to a Xevo TQ-S triple quadrupole mass spectrometer (Waters), and analysed using MassLynx software. Electrospray ionization was in positive ion mode: capillary voltage, 3.2 kV; cone voltage, 79 V; ion source temperature, 150 °C; collision energy, 45 V. Nitrogen and argon were used as the curtain and the collision gases, respectively. Reverse-phase HPLC was carried out at 30 °C using an Acquity UPLC BEH C18 1.7 μm, 1 × 50 mm column (Waters, cat no: 186002344). Buffers were 5% (v/v) ACN/0.1% (v/v) FA in water (buffer A) and 90% (v/v) ACN/0.1% (v/v) FA (buffer B). A gradient was run at 200 μL/min: 0–0.3 min, 5% B; 0.3–3 min, 5–100% B; 4–4.1 min, 100–5% B; 4.1–4.6 min, 5% B. Compounds were detected using multiple reaction monitoring using the following transitions: MitoB, 397 > 183; MitoP, 369 > 183, *d*_15_-MitoB, 412 > 191, *d*_15_-MitoP, 384 > 191. For each experiment standard curves were prepared in the appropriate biological material using known amounts of MitoB and MitoP, spiked with *d15*-MitoB and *d15*- MitoP internal standards and extracted in parallel with the samples.

### 2.3 Mouse interventions

For hyperoxia, litters of C57Bl6/J mice were exposed to 85% oxygen from P8 to P11, or to 75% oxygen from P7. Individual litters were not larger than 6 pups per litter, in order to ensure sufficient feeding and normal weight gain. Oxygen in the chamber was regulated by using a ProOx110 controller (BioSpherix). While litters were exposed constantly to hyperoxia, their nursing mothers were rested in room air for 2 h daily. We observed the mothers' health and investigated hyperoxia-induced lung injury by histological examination [34]. Age-matched litters were kept at room air as normoxic controls. For DMF treatment, *RPGR*^*−/−*^ mice from 3 months of age given drinking water with DMF (0.08% (w/v) methyl cellulose, 0.15% (w/v) DMF) [33]. The water was changed once a week.

### 2.4 Statistical analysis

Data was analysed using the GraphPad Prism software (Graphpad Software Inc.). Values are from individual eyes and means are presented ± SEM. To compare two unmatched groups unpaired two-tailed Student's t-test was used. ****p < 0.0001, ***p < 0.001, **p < 0.01, *p < 0.05.

## 3 Results

### 3.1 Measuring mitochondrial ROS formation in eyes *in vivo*

**Fig. 2:**
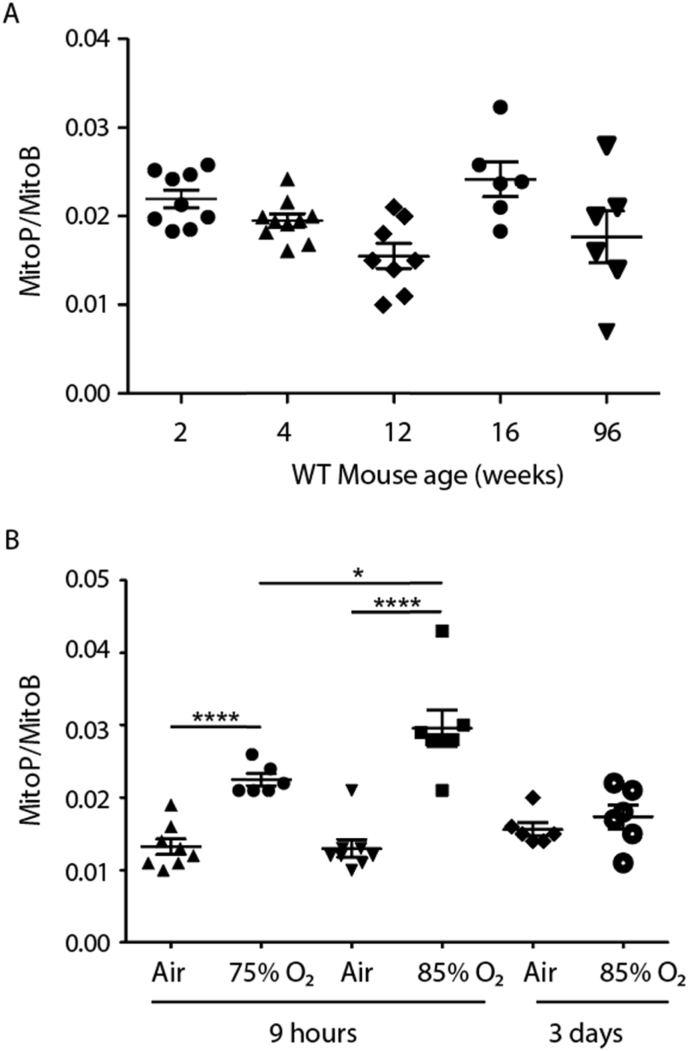
(A) Measurement of MitoP/MitoB following delivery of MitoB by injection into the posterior chamber of the eyes of C57Bl6/J mice. Mice were injected with 500 pmol MitoB in 1 μl PBS per eye and sacrificed 9 h post injection. Eyes were enucleated and snap-frozen until further analysis. Data are presented as individual measurements as well as means ± SEM. (B) MitoP/MitoB measurement following hyperoxia. C57Bl6/J pups together with their dams were placed under 75% oxygen (P7 pups) or at 85% O_2_ (P8 pups). MitoB was injected intravitreally into the pups' eyes before they were placed under hyperoxia for 9 h before being sacrificed, eyes removed and snap frozen for MitoP and MitoB extraction. Some p8 pups and dams were placed at 85% for 3 days, with the dams rested at room air for 2 h per day. After 3 days the pups were removed and their eyes were injected with MitoB as above, and then placed back under hyperoxia for a further 9 h before being sacrificed, eyes removed and snap frozen for MitoP and MitoB extraction. Data are presented as individual measurements as well as means ± SEM. An unpaired two-tailed Student's t-test was used to identify differences between groups. *p < 0.05, ****p < 0.0001.

MitoB has been previously used to measure H_2_O_2_ formation in living organisms such as flies and in mouse, heart, lungs and liver [25,26], but not the eye or specifically the retina. As MitoB does not readily cross the blood retina barrier, MitoB was injected intravitreally, with dose and timing experiments showing that injection of 1 μL of 500 μM MitoB followed by collection of the eyes after 9 h, allowed reliable MitoB and MitoP detection. To determine whether there are age-related changes to mitochondrial H_2_O_2_ levels, MitoP/MitoB ratios were measured in control (C57Bl6/J) mice up to 2 years of age. No increase in mitochondrial H_2_O_2_ formation was found with age (Fig. 2A). This shows that MitoB can be utilised to assess mitochondrial ROS production within the eye *in vivo*.

### 3.2 Generation of oxidative stress through hyperoxia

To determine whether MitoB responded to increased mitochondrial ROS formation *in vivo* within the eye, mice were exposed to increased external oxygen concentration. For this mouse pups were placed in a hyperoxia chamber at 75% or 85% O_2_ and the MitoP/MitoB ratio measured after 9 h under hyperoxia (Fig. 2B). After 9 h of hyperoxia the MitoP/MitoB ratio increased compared to normoxic, age matched controls (Fig. 2B). Interestingly, this acute elevation in mitochondrial ROS production was no longer observed after 3 days of hyperoxia at 85% O_2_ (Fig. 2B), either due to adaptation of the antioxidant defence system, or because of decreased oxygen delivery due to the vaso-obliteration that occurs during hyperoxia [[34], [35], [36]]. This shows that acute elevation of oxygen delivery to the retina increases mitochondrial ROS production and that analysis using MitoB enables changes in mitochondrial ROS within the eye to be assessed.

### 3.3 Elevated mitochondrial H_2_O_2_ production in models of retinal degeneration

We were next interested to see whether the increase in oxygen tension in the outer retina found in models of RP leads to increased mitochondrial ROS formation. To do this mouse models of RP with a range of severities and rapidity of onset were compared against age-matched wildtype mice.

**Fig. 3:**
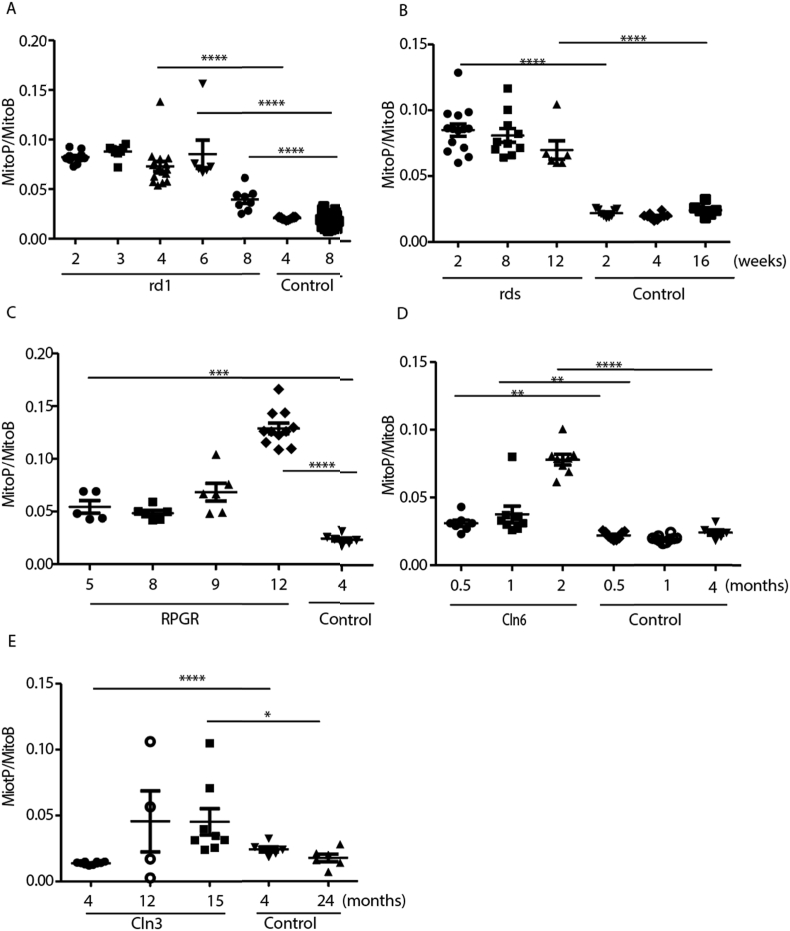
Measurement of MitoP/MitoB ratio in mouse models of retinal degeneration. Data are presented as individual measurements as well as means ± SEM. An unpaired two-tailed Student's t-test was used to identify differences between groups. *p < 0.05, **p < 0.01, ***p < 0.001 ****p < 0.0001. Mouse models used: A) rd1. B) rds. C) *RPGR*^*−/−*^. D) *Cln6*^*nclf*^. E) *Cln3*^*Δex7/8*^. Control indicates wild type mice.

The peak of rod degeneration in the rd1 mouse occurs at 21 days postnatal. The MitoP/MitoB level was already elevated at p14 and these levels were maintained up to week 6 post birth (Fig. 3A). MitoP/MitoB levels decreased 8 weeks post birth, most likely due to the almost complete loss of photoreceptors, removing the major source of H_2_O_2_. We observed a similar pattern of early mitochondrial ROS formation in the rds model, although the elevated ROS levels are maintained longer in the slower model (Fig. 3B). The rd1 and the rds models generated similar levels of mitochondrial ROS from early age during the degeneration of photoreceptors. In the slowest model of photoreceptor degeneration, the *RPGR*^*−/−*^ mouse, the MitoP/MitoB ratio was increased compared to age-matched wildtype controls 5 months postnatally, again preceding the peak in photoreceptor degeneration. However, the MitoP/MitoB levels were lower than in the rd1 and the rds mice. With increasing age, the MitoP/MitoB ratio reached similar values in the *RPGR*^*−/−*^ mouse as during the peak photoreceptor loss in the rd1 and rds models. As photoreceptor degeneration is incomplete over the lifespan of the *RPGR*^*−/−*^ mice we could not determine whether a decrease of mitochondrial ROS formation occurs later.

The *Cln6*^*nclf*^ mouse is a model of a lysosomal storage disorder that affects energy demanding neurons and leads to photoreceptor degeneration (Fig. 3D) [32]. As with the other models of photoreceptor degeneration, an increase in MitoP/MitoB ratios before the peak of degeneration was observed that increased with age (Fig. 3D).

A further mouse model of a lysosomal storage disorder is *Cln3*^*Δex7/8*^. While human patients with *CLN3* mutations experience photoreceptor loss as early as 6 years of age, the mouse model does not show significant photoreceptor loss over most of its lifespan [33]. There was no increase in the MitoP/MitoB ratio in *Cln3*^*Δex7/8*^ mice compared with age-matched wild type controls at 4 months, however at 1 year to 15 months of age, there was a small increase in mitochondrial ROS formation (Fig. 3E).

All these models showed that oxidative stress definitely precedes photoreceptor degeneration and that it increased with age and further loss of photoreceptors (Fig. 3A–E).

### 3.4 Lack of elevated mitochondrial H_2_O_2_ in loss of visual function models

**Fig. 4:**
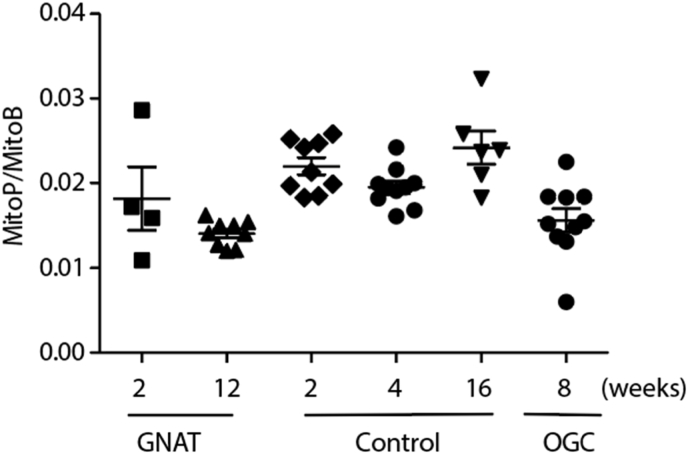
Measurement of MitoP/MitoB ratio in mouse models of loss of sight. Data are presented as individual measurements as well as means ± SEM.

The *Gnat1*^*−/−*^ and OGC mouse models show no loss of photoreceptors over their lifespan, but lack of specific proteins in the phototransduction cascade means that their vision is affected nonetheless. In these models no increase of mitochondrial ROS with age compared to age matched wildtype controls was observed (Fig. 4).

### 3.5 Reversal of elevated mitochondrial H_2_O_2_ by therapeutic interventions

**Fig. 5:**
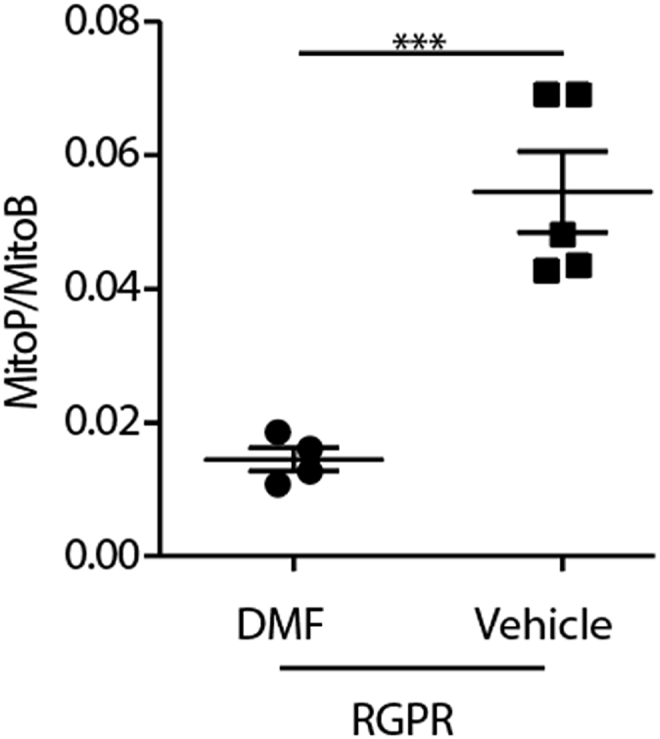
Measurement of MitoP/MitoB ratio in the *RPGR*^*−/−*^ mouse models of retinal degeneration. Following treatment with DMF for 5 months MitoB was injected intravitreally and mice maintained for 9 h before MitoP and MitoB extraction. Data are presented as individual measurements as well as means ± SEM. An unpaired two-tailed Student's t-test was used to identify differences between groups. ***p < 0.001.

As RP is a heterogeneous disease with many genetic causes, gene therapy is not always possible. Previously it has been shown that elevated expression of the redox regulator *Nrf2* has a positive effect on cone survival in rd1 mice [20]. To increase *Nrf2* activation of antioxidant and protective genes dimethylfumarate (DMF), which has shown promise in clinical trials for multiple sclerosis (MS) [37], was used. DMF has also been shown to prevent retinal ischaemia reperfusion injury, when administered as monomethyl fumarate i.p. to C57Bl6 mice [38]. *RPGR*^*−/−*^ mice were given DMF or vehicle control from 3 months of age for 2 months through their drinking water. DMF-treated *RPGR*^*−/−*^ mice showed a significant reduction in mitochondrial ROS after 2 months of treatment (Fig. 5). DMF may be a promising tool to prevent or postpone the death of photoreceptors.

## 4 Discussion

While oxidative stress has long been implicated in photoreceptor degeneration, previous measurements have focused on secondary markers [9]. Here we set out to measure mitochondrial H_2_O_2_ and peroxynitrite levels directly *in vivo* by using MitoB [26]. We established that MitoB could be used to measure mitochondrial ROS within the eye *in vivo*. While we cannot directly identify directly which cells within the eye are responsible for the increased levels of mitochondrial ROS, the photoreceptors are a likely source due to their high number of mitochondria.

We observed an increase in mitochondrial ROS during photoreceptor degeneration. There was an increase in mitochondrial ROS from the earliest tested time points in the fast degeneration models (rd1, rds), and the levels of mitochondrial ROS increased with age in the slower degenerating model (*RPGR*^*−/−*^). This increase in mitochondrial ROS preceded the actual photoreceptor degeneration. When *RPGR*^*−/−*^ mice were treated with DMF to upregulate *Nrf2* transcript levels MitoP/MitoB ratios did not increase compared to the vehicle control. Together these data point to the importance of oxidative stress and its control during PR degeneration. These findings, together with the increase in MitoP/MitoB ratios under high oxygen concentrations, point to raised oxygen supply increasing mitochondrial ROS formation within the outer retina, which in turn leads to photoreceptor cell death. We have also shown that supplying DMF to the drinking water reduces mitochondrial ROS. Further work is required to determine whether such treatments result in slowing of PR loss in mouse models and its potential to provide a generic treatment strategy for patients with RP.

## Author contributions

A.F.W, R.R.A., and M.P.M conceived the study; K.E.M., A. J. S., A.F.W, R.R.A., U.F.O.L. and M.P.M designed the experiments; K.E.M. and A. L. carried out experimental work; K.E.M., A. J. S., A.F.W, R.R.A., and M.P.M wrote the manuscript.

## Declaration of competing interest

UFOL is employee of F.Hoffmann-La Roche Ltd. The other authors declare that they have no known competing financial interests or personal relationships that could have appeared to influence the work reported in this paper.
